# Geometric and dosimetric evaluation for breast and regional nodal auto‐segmentation structures

**DOI:** 10.1002/acm2.14461

**Published:** 2024-08-02

**Authors:** Tiffany Tsui, Alexander Podgorsak, John C. Roeske, William Small, Tamer Refaat, Hyejoo Kang

**Affiliations:** ^1^ Department of Radiation Oncology Loyola University Chicago Stritch School of Medicine Maywood Illinois USA; ^2^ Department of Radiation Oncology Cardinal Bernard Cancer Center Maywood Illinois USA; ^3^ Department of Radiation Oncology University of Rochester Medical Center Rochester New York USA

**Keywords:** AI‐algorithm, Auto‐contour, auto‐segmentation, breast radiation treatment

## Abstract

The accuracy of artificial intelligence (AI) generated contours for intact‐breast and post‐mastectomy radiotherapy plans was evaluated. Geometric and dosimetric comparisons were performed between auto‐contours (ACs) and manual‐contours (MCs) produced by physicians for target structures.

Breast and regional nodal structures were manually delineated on 66 breast cancer patients. ACs were retrospectively generated. The characteristics of the breast/post‐mastectomy chestwall (CW) and regional nodal structures (axillary [AxN], supraclavicular [SC], internal mammary [IM]) were geometrically evaluated by Dice similarity coefficient (DSC), mean surface distance, and Hausdorff Distance. The structures were also evaluated dosimetrically by superimposing the MC clinically delivered plans onto the ACs to assess the impact of utilizing ACs with target dose (Vx%) evaluation.

Positive geometric correlations between volume and DSC for intact‐breast, AxN, and CW were observed. Little or anti correlations between volume and DSC for IM and SC were shown. For intact‐breast plans, insignificant dosimetric differences between ACs and MCs were observed for AxN_V95%_ (*p* = 0.17) and SC_V95%_ (*p* = 0.16), while IMN_V90%_ ACs and MCs were significantly different. The average V95% for intact‐breast MCs (98.4%) and ACs (97.1%) were comparable but statistically different (*p* = 0.02). For post‐mastectomy plans, AxN_V95%_ (*p* = 0.35) and SC_V95%_ (*p* = 0.08) were consistent between ACs and MCs, while IMN_V90%_ was significantly different. Additionally, 94.1% of AC‐breasts met ΔV95% variation <5% when DSC > 0.7. However, only 62.5% AC‐CWs achieved the same metrics, despite AC‐CW_V95%_ (*p* = 0.43) being statistically insignificant.

The AC intact‐breast structure was dosimetrically similar to MCs. The AC AxN and SC may require manual adjustments. Careful review should be performed for AC post‐mastectomy CW and IMN before treatment planning. The findings of this study may guide the clinical decision‐making process for the utilization of AI‐driven ACs for intact‐breast and post‐mastectomy plans. Before clinical implementation of this auto‐segmentation software, an in‐depth assessment of agreement with each local facilities MCs is needed.

## 1 INTRODUCTION

Recent advancements in deep learning (DL) have led to substantial development of DL‐driven auto‐segmentation (AS) algorithms, which have been rapidly advancing to expedite radiation treatment planning compared to the time‐consuming manual segmentation process. Studies have shown that AS substantially increases the efficiency of the treatment planning process^1^, ^2^, ^3^, ^4^ and helps to reduce inter‐observer variability in target and organs‐at‐risk (OAR) delineation. ^5^, ^6^, ^7^, ^8^ Although AS target volumes have emerged as an active research area with promising results,^3^, ^4^, ^5^, ^9^, ^10^, ^11^ AS has been more commonly implemented for OAR delineation for various disease sites.^12^, ^13^, ^14^, ^15^, ^16^, ^17^ Accurate AS of both target structures and OAR is necessary for adaptive radiotherapy, as the adaptive treatment planning process should be completed within a couple of minutes following image acquisition while the patient is in the treatment position.^18^ However, AS faces challenges in its performance and clinical use especially for target definition considering the serious impact of errors in radiation treatment,^19^ which may lead to partial or complete miss in targeting tumors and over‐irradiating surrounding healthy tissue.

With the advancement in DL‐driven AS, which has shown greater accuracy compared to atlas‐based AS methods and has become the mainstream approach ^7^, ^20^, ^21^, ^22^, ^23^, ^24^ multiple commercial artificial intelligence (AI) or DL‐driven algorithms became available for routine clinical use.^25^, ^26^, ^27^, ^28^, ^29^, ^30^, ^31^, ^32^, ^33^ Recently, several commercial systems have implemented full automation of target contours for breast cancer treatment in addition to OARs (Therapanacea, MVISION, Limbus AI & Radformation). Despite the potential benefits of AS in streamlining the labor‐intensive breast and nodal target delineation, there still remain challenges to overcome, particularly concerning defining the “ground truth” for target segmentation. The lack of a concrete definition for the ground truth in target segmentation may be due to variations between physicians based on their experience levels, contouring styles, or clinical protocols used as guidelines for target contours.^34^, ^35^, ^36^, ^37^ In addition, commercial algorithms pose different challenges compared to in‐house DL algorithms since the software vendors typically do not provide the users with access to the patient data used to train the algorithm and the training process of the algorithm is not disclosed to the users. Nonetheless, many of them employ the U‐Net architecture, provide AS for organs at risk of various disease sites, including the brain, head and neck, thorax, abdomen, and pelvis.^38^ Therefore, it is essential for users to perform comprehensive assessments using local patient data to evaluate the effectiveness, accuracy, and limitation of the algorithm and to identify when and how algorithms fail to generate accurate segmentation prior to clinical deployment.^33^

Prior to the clinical deployment of commercial algorithms, it is crucial to thoroughly assess both the geometric and dosimetric impacts of utilizing auto‐segmented target structures compared to physician‐drawn target structures. The assessments determine whether the accuracy and reliability of the algorithms are clinically acceptable for a particular clinic, which may have patient characteristics different from other hospitals or those of the cohorts used to train the algorithms. Currently, there are very few studies investigating the impact of using AS algorithms for breast and regional nodal structures, as it is still relatively new.^31^ As a consequence, there is a lack of guidelines on how to clinically implement the commercial AS algorithms of these target structures, and little is known about their performance.

Our goal is twofold. First, a commercial DL‐driven algorithm, AutoContour (RADformation, USA)^39^, ^40^ is being validated retrospectively with geometric and dosimetric parameters using patient data from our institution. Second, the study aims to determine the geometric parameters of the breast and regional node target structures and physician contouring styles that correlate with consistent dosimetric distribution between AS using AutoContour and the gold standard of physician manually‐segmented contours (MC).

We investigate how target volumes and target geometries, such as width and length, affect geometric accuracy. Our evaluation provides valuable insights into the dosimetric impacts of utilizing AS breast and regional nodal structures and may lead to the guidelines for the adoption of AS in the clinic. This study serves as a paradigm on how to evaluate DL algorithms for other target structures as they become available.^41^, ^42^ Furthermore, this study pioneers as a blueprint for assessing AS technology for disease sites, other than breast treatment volumes, as clinics implement AS with new structures.

## 2 METHODS

### 2.1 Patient data collection and processing

The data collection process includes population all consecutive breast and chestwall (CW) patients that was treated at our institution between January 2021 and December 2022, excluding treatment plans that do not fit our study criteria. The patient selection criteria include a prescription dose of 5040 cGy (180 cGy per fraction), no re‐irradiation cases, no intensity modulated radiotherapy (IMRT) or volumetric modulated arc therapy (VMAT), and no bilateral breast/CW cases. In this Institutional Review Board‐approved study, the planning data for 66 breast cancer patients (34 intact breast, 32 post‐mastectomy chest wall) were utilized. All patients received 3D conformal radiotherapy (3DCRT). Prior to treatment, all patients received a CT simulation (Siemens Definition AS CT simulator Siemens Healthineers, Munich, Germany) in the supine position using a technique with 120 kVp, 198 mAs, and 3 mm slice thickness. The structures of interest for intact‐breast treatment included “Breast_Eval”, total axillary nodes (AxN), internal mammary nodes (IMN), and supraclavicular lymph nodes (SC). “Breast_Eval” was defined as the breast structure cropped 5 mm from the skin and hereinafter known simply as breast. AxN included all three axillary nodal levels. The structures of interest for post‐mastectomy radiation treatments included AxN, IMN, SC, and “Chestwall_Eval”, which was the CW structure cropped 5 mm from the skin and hereinafter known simply as CW. As part of the standard treatment planning process, the structures of interest were manually delineated in our treatment planning system, Eclipse (Varian Medical Systems, Palo Alto, CA, USA) by one of two attending radiation oncologists (MD1, *n* = 27, MD2, *n* = 39). The two radiation oncologists possess extensive experience in treating patients with breast cancer. One MD has 30 years of experience, while the other MD has 20 years of experience. Their extensive backgrounds are underscored by numerous publications focusing on breast cancer and the intricacies of breast contours. Radiation treatments were planned with four fields where the breast and CW target volumes were treated with two tangentially‐opposed fields, and the regional nodes were treated with two anterior/posterior oblique fields for a standard mono‐isocentric 3DCRT.

### 2.2 Target auto‐segmentation software

The processing of the AS target volumes was performed within the Radformation AutoContour software (Radformation, New York, USA), which is AI‐based, and functioned as a plug‐in within Eclipse. Radformation AutoContour was chosen for this study because it is the only AS tool readily available at our specific institution and works seamlessly with our current treatment planning system, Varian Eclipse. The volumes for the breast/CW, AxN, SC, and IMN were retrospectively generated within AutoContour and exported back to Eclipse. No manual modification was performed on the automatically‐segmented‐contours (AC). The entire automatic contouring process took 1−2 min per patient.

The ACs generated via the AutoContour software were compared against the corresponding physician's MC structures. Different quantitative metrics were used to geometrically and dosimetrically evaluate the similarity between the AC and MC structures.

### 2.3 AutoContour geometric evaluation

The volumes of AC in cubic centimeters measured within Eclipse were compared with the corresponding MC volumes. The MC and AC volumes were then transferred to Velocity Oncology Imaging Informatics System (Varian Medical Systems, CA, USA) for the computation of the Dice similarity coefficient (DSC), mean surface distance (MSD), and Hausdorff Distance (HD) for each patient.

DSC is a metric that assesses the spatial overlap of two sets and ranges from 0 to 1, with 1 indicating perfect overlap and 0 indicating no overlap. The DSC is given by:

(1) $$\text{DSC}=\frac{2\times \left|X\cap Y\right|}{\left|X\right|+\left|Y\right|}$$

where *X* is the structure of interest (i.e., AC) and Y is the ground truth (i.e., MC).

MSD and HD are defined respectively as:

(2) $$\mathrm{MSD}=\mathrm{mean}\left(d\left(X,Y\right),\;d\left(Y,X\right)\right)$$

(3) $$\mathrm{HD}=\max\left(d\left(X,Y\right),\;d\left(Y,X\right)\right)$$

where d(X,Y) and d(Y,X) are the forward and backward distances, respectively from *X* to Y. The MSD and HD metrics measured the mean and maximum spatial distance, respectively, between two structure sets, where perfect overlap would yield a distance of 0 cm. The correlation of these metrics on MC and AC target volume was assessed via the Pearson correlation coefficient *r*.

To determine if the performance of AC was physician‐agnostic, we sorted the data per physicians (i.e., MD1 and MD2) and performed a subgroup analysis accordingly. As further investigation to determine if the performance of AC can be predicted by geometric parameters, the length for each three‐dimensional direction was analyzed. These parameters may provide reliable metrics in predicting whether certain structure types or sizes would achieve a better resemblance between AC and MC.

### 2.4 AutoContour dosimetric evaluation

Using the clinically delivered treatment plan, a radiation dose parametric assessment was carried out comparing target coverage considering MC and AC target structures. The dosimetric parameter selected for the breast/CW, AxN, and SC was the V_95%_, which represents the percentage of the planning target volume (PTV) that received at least 95% of its prescribed dose. For the IMN, V_90%_, the percentage of PTV that received at least 90% of its prescribed dose, was selected due to the volume and location of the structure with respect to the treatment field edge to spare various OARs including the humeral head.^43^, ^44^ The dosimetric parameters were selected as part of the plan quality evaluation for each specific anatomic site.^45^, ^46^ The absolute value of the difference in the V_x%_ or V_x%_ for the MC and AC structures was computed and reported as a |ΔV_x%_| and given by:

(4) $$\Delta Vx\%=\frac{MC_{Vx\%}-AC_{Vx\%}}{MC_{Vx\%}}\times 100\%$$

where $MC_{Vx\%}$ and $AC_{Vx\%}$ are the PTV percentage of MC and AC, respectively, that received at least *x*% of its prescribed dose. *x*% is 90% for IMN, and 95% for breast/CW, AxN, and SC. Perfect overlap of the two contours would lead to no difference in the target coverage. We assessed the correlation of |ΔVx%| with the volume of the target organ being considered. Similar to the geometric assessment, the correlation was assessed using the Pearson correlation coefficient *r*.

The AC target dose coverage (V90% for IMN and V95% for breast/CW, AxN, and SC) for intact‐ and post‐mastectomy‐breast plans were assessed using a one‐tail t‐test. Dosimetric‐geometric correlation of ACs was evaluated using the target dose difference between ACs and MCs (ΔVx%) versus DSC. A DSC metric of ≥0.7 is often considered a satisfactory volume match.^47^, ^48^, ^49^, ^50^

**FIGURE 1:**
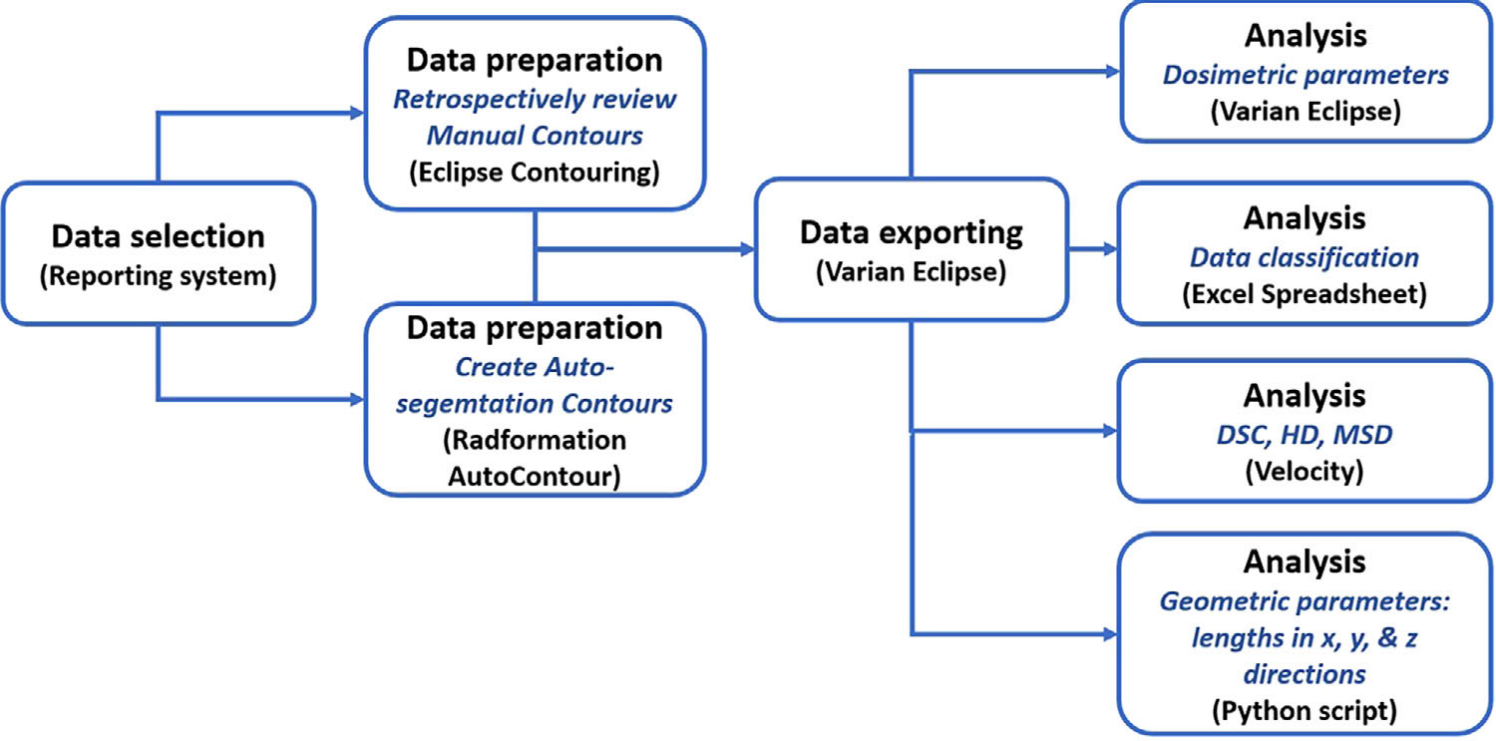
Overall study workflow that includes data selection in AURA, data preparation in Eclipse Contouring and Radformation Auto Contour, data export in Varian Eclipse, and data analysis in Eclipse, Velocity, Excel, and Python. AURA, Aria reporting system.

The overall workflow of the study is shown in Figure 1, which includes, data selection, data preparation, and data exporting prior to data analysis. The analysis includes geometric evaluations (performed in Varian Velocity and Python script) and dosimetric evaluations (performed in Varian Eclipse). Data are evaluated and classified using a detailed Excel spreadsheet.

## 3 RESULTS

### 3.1 Geometric evaluation

**FIGURE 2:**
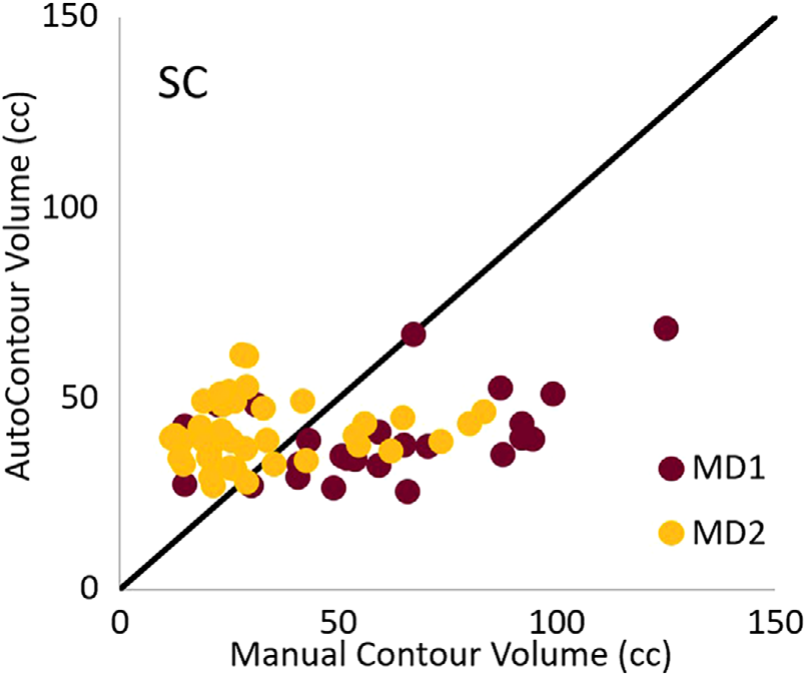
Physician‐dependent performance of AC for the SC. The volumes of MC are systematically larger than the AC volumes for MD1, which is not observed for MD2. The identity line is plotted in black. AC, automatically‐segmented contours; MC, manually‐segmented contours; SC, supraclavicular nodes.

We found a large influence on geometric accuracy dependent on the target structures considered (Table 1). The breast structure demonstrated the best segmentation performance assessed via DSC, HD, and MSD averaged over our collected dataset. Mastectomy status had an impact, in that AC structures were significantly less accurate for the CW compared with the breast. Based on the DSC for different lymph node structures, AC had good agreement with MC for the AxN, some agreement with the SC, and little agreement with the IMN. Looking solely at volume agreement, good physician‐agnostic performance of AC was observed in all but the SC (Figure 2). Looking closer at the SC, MD1 systematically contoured larger than ACs, whereas the MCs from MD2 did not exhibit this characteristic.

**TABLE 1:** Geometric accuracy quantified with Dice similarity coefficient (DSC), Hausdorff distance (HD), and mean surface distance (MSD) averaged over our entire patient dataset.

	Breast	CW	AxN	SC	IMN
DSC	0.85 ± 0.06	0.71 ± 0.20	0.70 ± 0.09	0.54 ± 0.14	0.33 ± 0.17
HD (mm)	38.07 ± 12.59	38.49 ± 25.76	36.3 ± 14.74	41.00 ± 24.01	41.81 ± 16.00
MSD (mm)	4.32 ± 1.70	6.90 ± 8.52	5.22 ± 2.27	9.69 ± 6.29	8.99 ± 5.38

*Note*: Superior segmentation performance was seen with the intact breast relative to the post‐mastectomy chestwall (CW). The axillary node (AxN) were the most successfully auto‐contoured structure of the nodal structures.

**FIGURE 3:**
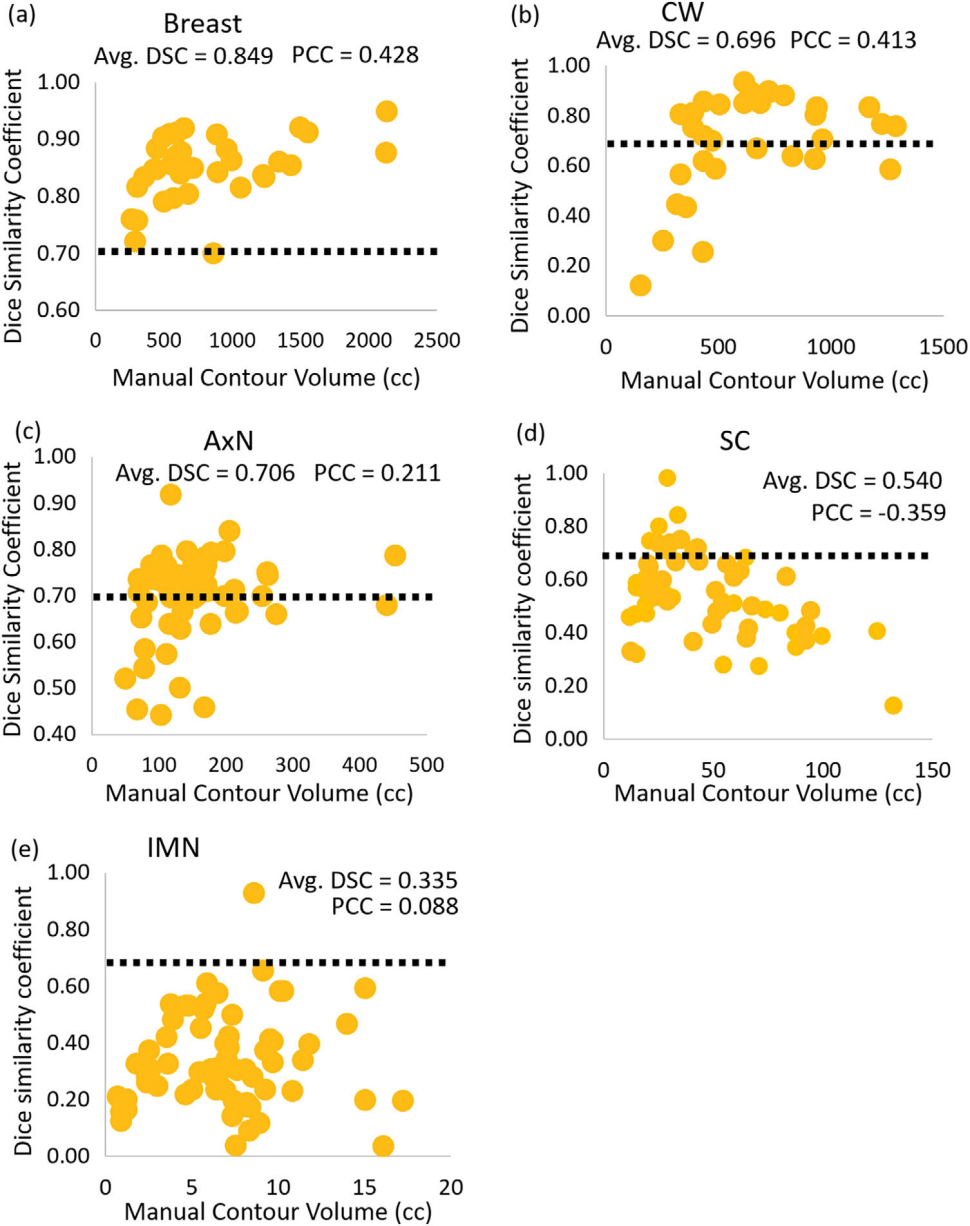
DSC versus the MC volumes in cubic centimeters for the considered target structures. Correlation is denoted by the PCC. Positive correlation between the two quantities was observed for (a) the breast, (b) CW, and (c) AxN. Anti‐correlation and weak correlation were observed for the (d) SC and (e) IMNs, respectively. AxN, axillary nodes; CW, chestwall; DSC, Dice similarity coefficient; IMN, internal mammary nodes; MC, manually‐segmented contour; Pearson correlation coefficient; SC, supraclavicular nodes.

When considering the correlation between contour volumes and a geometrically accurate AC based on DSC (Figure 3), a positive correlation was found between the MC volume and the DSC for the breast (*r* = 0.428), CW (*r* = 0.413), and AxN (*r* = 0.211). Little positive or even anti‐correlation was observed for the IMN (*r* = 0.088) and SC (*r* = −0.359) respectively.

**FIGURE 4:**
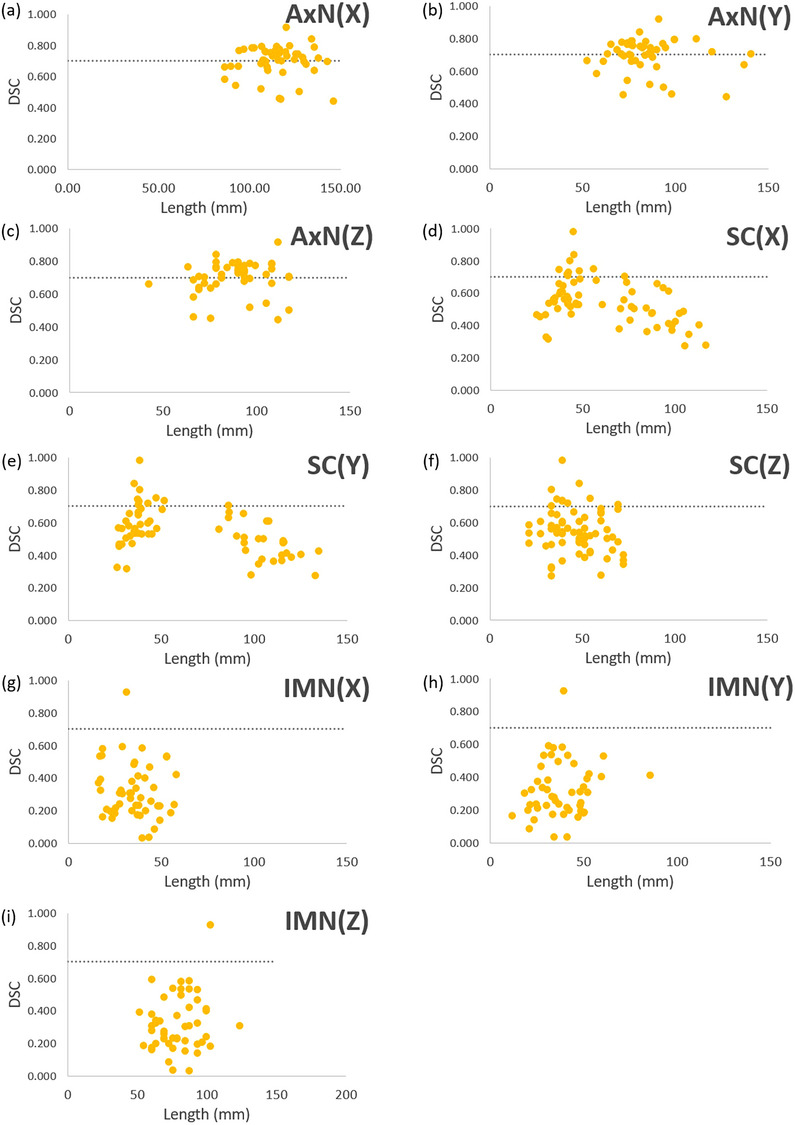
DSC versus the lengths in three dimensions of target structures. (a)–(c) AxN in *x*‐, *y*‐, and z‐directions are denoted by AxN(*X*), AxN(*Y*), and AxN(*Z*), respectively. (d)–(f) SC in *x*‐, *y*‐, and z‐directions are denoted by SC(*X*), SC(*Y*), and SC(*Z*), respectively. (g)–(i) IMNs in *x*‐, *y*‐, z‐directions are denoted by IMN(*X*), IMN(*Y*), and IMN(*Z*), respectively. AxN, axillary nodes; DSC, Dice similarity coefficient; IMN, internal mammary nodes; SC, supraclavicular nodes.

To provide a geometrical metric in predicting the performance of AC, the lengths in three directions of each structure were tabulated against the corresponding DSC data as shown in Figure 4. There is minimal correlation between the length and DSC in most structure sets. Correlations can be observed in the *x*‐ and *y*‐dimensions of the SC (Figure 4d,e), which represent the left/ right and superior/ inferior orientation respectively. When SC are over 70 mm in *x*‐ and/or *y*‐ dimensions, 3.8% of the SC structures have a DSC of higher than 0.7. When SC structures are less than 70 mm in *x*‐ and/or *y*‐dimensions (Figure 4d,e, respectively), 34.6% of the SC structures have a DSC of higher than 0.7. Therefore, upon review of the AC structures, SCs > 70 mm in *x*‐ and/or *y*‐directions have no correlation with MCs. There might be greater confidence that SCs with less than 70 mm in *x*‐ and/or *y*‐dimensions would have close resemblances with MCs compared to the larger SCs (Figure 4d,e).

### 3.2 Dosimetric evaluation

As shown in Table 2, for intact‐breast plans, insignificant differences between ACs and MCs were observed for AxN_V95%_ and SC_V95%_, while IMN_V90%_ ACs and MCs were significantly different. The average V95% for the breast MCs and ACs were comparable but statistically different. The results of CW plans (Table 3) are similar to that of breast plans. For CW plans, AxN_V95%_ and SC_V95%_ were also consistent between ACs and MCs, while IMN_V90%_ was significantly different. The mean values of CW_V95%_ are comparable with no statistical difference.

**TABLE 2:** Mean and standard deviation (SD) of automatically‐segmented contour (AC) and manually‐segmented contour (MC) with *p*‐values between MC and AC structures for intact‐breast plans.

Intact Breast plans (*n* = 34)					
		Mean	SD	*p*(*T* < = *t*) one‐tail	Plans with DSC > 0.7 and ΔV95/90 < ± 5%
**Breast V95%**	MC	98.36	1.95	0.014	94.12%
	AC	97.18	2.38		
**AxN V95%**	MC	95.53	6.58	0.167	67.65%
	AC	93.92	7.01		
**SC V95%**	MC	91.01	18.37	0.083	14.71%
	AC	84.61	19.26		
**IMN V90%**	MC	84.61	19.26	0.000	0.00%
	AC	61.66	27.58		

*Note*: V95% and V90% represent the percentage of the planning target volume (PTV) that received at least 95% and 90%, respectively, of its prescribed dose. The axilliary nodes, supraclavicular nodes, and internal mammary nodes are denoted by AxN, SC, and IMN, respectively.

**TABLE 3:** Mean and standard deviation (SD) of automatically‐segmented contour (AC) and manually‐segmented contour (MC) with *p*‐values between MC and AC structures for post‐mastectomy chestwall plans.

Post‐mastectomy breast plans (*n* = 32)					
		Mean	SD	*p*(*T* < = t) one‐tail	Plans with DSC > 0.7 and ΔV95/90 < ± 5%
**CW V95%**	MC	96.90	2.91	0.191	62.50%
	AC	96.04	4.71		
**AxN V95%**	MC	93.31	13.09	0.352	56.25%
	AC	92.07	12.82		
**SC V95%**	MC	91.18	17.43	0.084	9.38%
	AC	84.92	18.49		
**IMN V90%**	MC	83.46	29.35	0.001	3.13%
	AC	61.31	26.22		

*Note*: V95% and V90% represent the percentage of the planning target volume (PTV) that received at least 95% and 90%, respectively, of its prescribed dose. The CW, axilliary nodes, supraclavicular nodes, and internal mammary nodes are denoted by CW, AxN, SC, and IMN, respectively.

**FIGURE 5:**
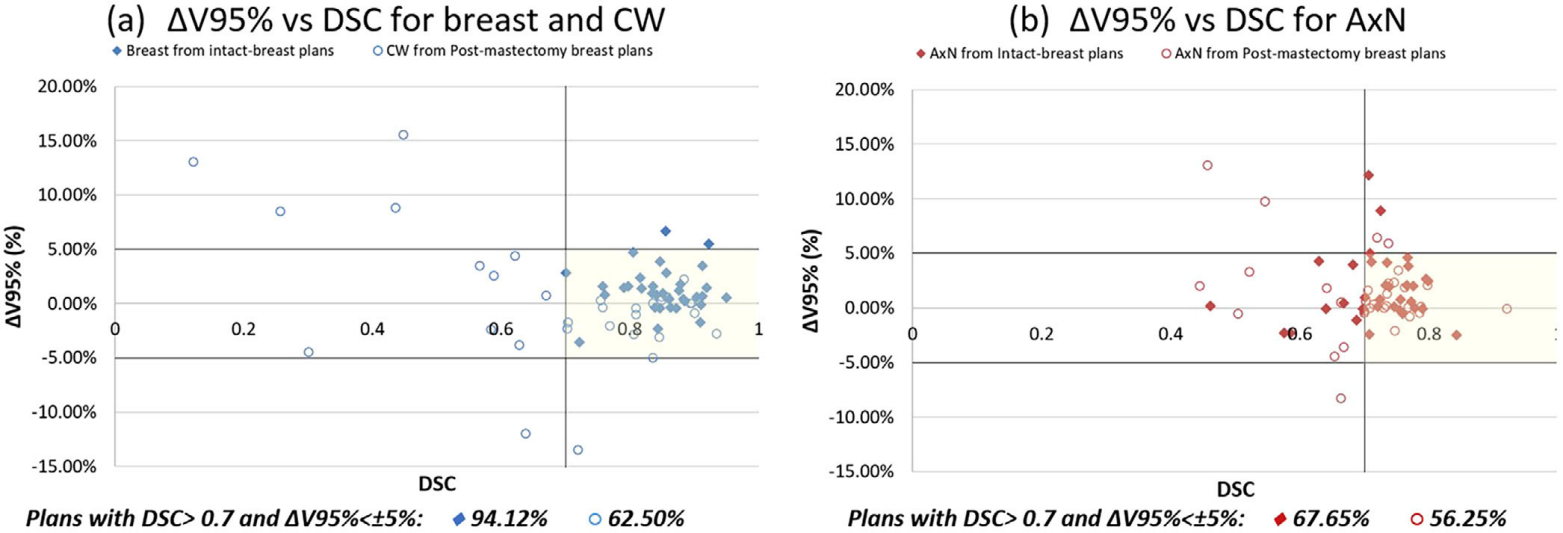
ΔV95% versus DSC for (a) breast and CW and (b) AxN in intact‐breast and post‐mastectomy breast plans. AxN, axillary nodes; CW, chestwall; DSC, Dice similarity coefficient.

Figure 5a shows 94.1% breast ACs meeting both ΔV95% < ± 5% and DSC > 0.7, while only 62.5% CW ACs meet this metric, despite AC‐CW_V95%_ being statistically insignificant (Tables 2 and 3). For AxN, a low percentage of plans (67.65% of breast and 56.25% of CW plans) meet the DSC > 0.7 and ΔV95% < ± 5% metric (Figure 5b). Similarly for SC, even a lower percentage of plans (14.71% of breast and 9.38% of CW plans) meet the DSC > 0.7 and ΔV95% < ± 5% metric (graph not shown). For IMN, which is small and on the radiation field edge, it shows a significant difference between MC and AC in V90% coverage for both breast and CW plans (Table 2). All except for one IMN case had DSC values < 0.7 (graph not shown).

**FIGURE 6:**
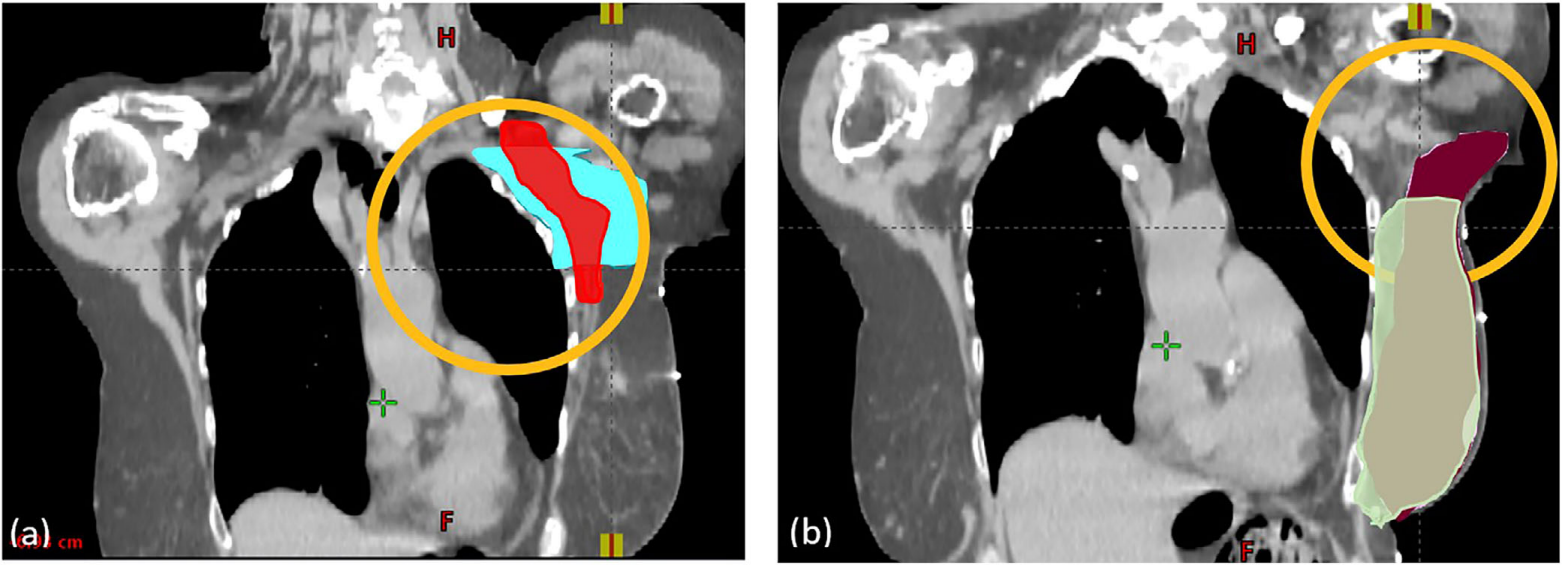
Two examples of out‐of‐plane discrepancies between MC and AC. (a) Out‐of‐plane discrepancies between MC (red) and AC (cyan) of AxN; (b) Out‐of‐plane discrepancies between MC (green) and AC (burgundy) of breast. These cases would have reduced dosimetric agreement in terms of the V95% relative to in‐plane accuracy, as the field aperture for a 3DCRT would be defined by the superior and inferior borders of the target. V95% represents the percentage of the PTV that received at least 95% of its prescribed dose. 3DCRT, 3D conformal radiotherapy; AC, automatically segmented contour; AxN, axillary node; MC, manually‐segmented contour; PTV, planning target volume.

Considering the dosimetric results, the use of a 3DCRT technique for these treatments is important to note as it defines gradient of the prescription dose falls off from the target contour. There is probably less demand for an accurate contour in‐plane for 3DCRT compared to more conformal plan techniques such as static gantry IMRT or VMAT. The out‐of‐plane dose discrepancies are essential (Figure 6), as the field aperture will be defined based on the superior/inferior extent of the targets.

## 4 DISCUSSION

This study investigates the clinical performance of a commercial DL‐driven AS software for the breast, CW, and regional nodal structures for external beam radiation therapy treatment planning for breast cancer. While there are studies previously published considering the geometric and dosimetric difference between the manual‐ and auto‐contouring process for head and neck,^51^ pelvic, and abdomen treatments,^49^ there has yet to be any guidance on incorporating DL‐driven segmentation tools for radiotherapy of the breast/CW and regional nodal structures.

This study is the first investigation, to the best our knowledge, that performs a clinically relevant assessment of the clinic‐specific performance of the Radformation AutoContour software in the anatomical targets of the breast/CW and regional nodal structures using local or clinical‐specific patient data. Our study found that for certain structures, particularly intact‐breast and AxN, a commercial DL‐driven AS tool trained with an external dataset was able to achieve good physician‐agnostic segmentation performance without much secondary manual edits and correlated with good dosimetric performance. For other structures, specifically the CW and SC, the AC provided a reasonable starting point but would require manual editing for clinical acceptability. Our results showed that there was limited geometric and dosimetric agreement considering the AC of the IMNs and that the AC model will require more vendor tuning. IMNs were more geometrically and dosimetrically sensitive to contour variations, possibly because of the small volume and its location near field edges.

It is important to note that the outcome of this study is dependent on several factors, including the vendor's method in data training of the DL‐driven tool, the type of data used in each model's development, whether both intact‐breast and post‐mastectomy CW patients’ data were included in the data training, and definition of the ground truth for the DL‐driven software. There was some physician dependence noted in our clinic in the SC, where one of the attending MDs utilized a different contouring atlas than the other MD and the accuracy of AC SC depends on the length in the lateral (*x*) or superior/ inferior (y) directions. Our results show that shorter SCs (< 70 mm) have better agreement between AC and MC. This is an example where a generalized model that agrees with all observers may not be possible for the current AI software. Therefore, internal review and local validation should be performed for all AC structures prior to clinical implementation.

Considering the correlation between the target volumes and |ΔV_95%_|, there are two possible interpretations of the anti‐correlation. First, structures with larger volumes result in more comparable MCs and ACs, and better spatial overlap of the AC results with that from the MC leads to more dosimetric agreement. This is supported by the results seen from Figures 2, 3, 4, 5, which showed a positive correlation between DSC and the MC volume. The second is that larger organs have a larger field size considering the 3DCRT treatment technique utilized in this patient cohort. This may have led to a further reduction in the demands for an accurate in‐plane contour, relative to smaller structures with more selective aperture openings.

This study does have limitations that should be considered. Our clinical site only performs breast, CW, and regional nodal treatments with a 3DCRT technique, hence our current study only includes 3DCRT breast and CW treatments. In the future, a larger multi‐institutional data set can be included to study plans in other institutions with intensity‐modulated techniques that produce a more conformal dose around the targeted. Additionally, we only considered 66 patients in our dataset. It is likely that there are unique cases (e.g., unique anatomy, clinical history, or pathological findings) not included in the trained model that may challenge the segmentation software more, leading to more variation in geometric and dosimetric performance for breast cancer patients. These unique cases may require volume adjustments that deviate from a standard protocol based on clinical judgment with an individualized treatment approach. With a larger dataset, we will be able to subcategorize data factors (such as gender, age, imaging and planning protocols) to enrich the significance of the study results.

Throughout this study, the authors encountered various challenges, encompassing data selection, collection, preprocessing, and evaluation. During the process of data selection and collection, we deliberately excluded data that might bias the results, considering factors such as prescription, prior irradiation, treatment modality (3D vs. IMRT), and cases involving bilateral conditions. However, we acknowledge the potential benefit of incorporating such data categories in future studies if they become adequately represented in our dataset. Additionally, the challenge of acquiring sufficient data, particularly in the realm of AI research, is a common obstacle. At our institution, it took a considerable amount of time to accumulate a diverse dataset of breast cases. To address these challenges, we intend to collaborate with other radiation oncology teams to incorporate multi‐institutional data in future research endeavors.

In the course of this study, significant effort was dedicated to data preprocessing to ensure data validity and consistency. Notably, discrepancies arose regarding the definition of the CW structure between the AutoContour software and the protocol utilized by one of our MDs. Consequently, we manually verified and regenerated chest wall contours cropped 5 mm from the skin to align with the AutoContour protocol. These discrepancies underscore the importance of standardized guidelines and protocols across institutions to mitigate such variations resulting from evolving clinical practices.

There were challenges in identifying meaningful metrics to evaluate the accuracy of the AutoContour software. The results are dependent on various factors, including the contour size, shape, and spatial relationship to the target structures. Consequently, our findings offer comprehensive guidance on the effectiveness of individual structures, delineating those that statistically perform well and those that do not. The parameters we opted for in this paper are widely used and easily comprehensible for most readers. Additionally, to ensure efficiency and consistency in the study workflow, we chose to utilize software already integrated into our institution's routine clinical operations. The metrics we utilized are accessible and generated through commercial software, Velocity (Varian, Siemens Healthineers, CA, USA). Furthermore, this streamlined workflow facilities seamless collaboration with other institutions equipped with Varian Velocity and potentially expands our dataset size in the future. We are planning to incorporate more inclusive metrics such as mean HD, minimum HD, volume correlation, relative absolute volume difference, and specificity in our future study using larger datasets from multi‐institutions.

To mitigate these limitations, we have implemented several solutions, including maintaining a detailed spreadsheet to track all cases, adhering to standardized contouring protocols, and implementing cross‐checking procedures to identify and rectify data outliers stemming from human errors. Furthermore, we plan to establish proper quality assurance procedures for target volume auto segmentation in future studies.

This study can be extended in multiple promising directions for future work. Firstly, re‐planning the 3DCRT treatments on the AC targets and assessing how the dose is delivered to the MC targets and surrounding OARs relative to the clinically delivered plan might provide a more comprehensive perspective as to the true dosimetric impact of AC in this anatomical site. Secondly, performing a time assessment of AC with physician adjustments of each target structure and comparing that with the time for physician contouring from scratch would yield the potential workflow impact of AC use.^52^ Furthermore, the continuation of this study can be changed from a retrospective approach to a prospective approach to evaluate manual contours that used AI tools as a supportive tool by enrolling patient datasets in clinical trials.

Moving forward, studies such as these will be important for implementing more AI‐based tools into the Radiation Oncology clinic. NRG Oncology consensus papers^53^ mention the future use of AI, not only for AS, but for other workflow improvements during image registration, treatment planning, and even radiation delivery. As more clinics move in the direction of AI‐based tools, there will unavoidably be questions pertaining to how these tools should be properly assessed and implemented following purchase. This study presents a framework for this in the context of AS; however, this can be expanded to the broader scope of the implementation of AI‐tools to Radiation Oncology in general. The tool must be assessed using local patient data, practice habits, styles, and workflows, with proper oversight of all members of the Radiation Oncology team. With all of these steps in place, we as a field can move forward into the next generation of AI‐assisted Radiation Oncology, to the end of providing better care to our patients.

In summary, we present a clinically‐relevant performance analysis of a commercial AS tool, Radformation AutoContour software, for the segmentation of the breast/CW and regional nodal structures. This tool has the capacity to greatly improve the throughput and workflow of radiation treatment planning for this anatomical site. However, organ‐specific assessments of the tool are recommended to gain an understanding of the segmentation agreement with the local contouring physicians and how practice differences may lead to agreement or otherwise.

## 5 CONCLUSION

The AC breast structure was geometrically and dosimetrically similar to physicians’ MCs, which could be used for treatment planning without modification for our clinic. AC AxN and SC may require some manual adjustments. Careful review should be performed for AC CW before treatment planning. Our results show that AC IMN is not usable yet in a clinic without careful reviewing and extensive editing. The SC MC and AC volumes agreed better for smaller lengths in both lateral and superior/ inferior directions. Thus, the findings of this study may guide the clinical decision‐making process for the utilization of DL‐driven AS for intact‐ and post‐mastectomy breast plans. Since AI contouring algorithms are specific to the training data set and various protocols, practitioners, and unique patient anatomies, local validation is essential for each clinic prior to the implementation of any AI contouring tools in a clinical setting.

## AUTHOR CONTRIBUTIONS

The authors confirm their contribution to the paper as follows: Original idea: Hyejoo Kang. Study conception and design: Hyejoo Kang, Alexander Podgorsak, Tiffany Tsui. Data Collection: Hyejoo Kang, Alexander Podgorsak, Tiffany Tsui. Analysis and interpretation of results: Hyejoo Kang, Alexander Podgorsak, Tiffany Tsui, John C. Roeske, William Small Jr., Tamer Refaat. Project supervision: Hyejoo Kang, John C. Roeske, William Small Jr., Tamer Refaat. Draft manuscript preparation: Hyejoo Kang, Alexander Podgorsak, Tiffany Tsui. All authors reviewed the results and approved the final version of the manuscript.

## CONFLICT OF INTEREST STATEMENT

The authors have no affiliations with or involvement in any organization or entity with any financial interest.
